# Learning equity requires more than equality: Learning goals and achievement gaps between the rich and the poor in five developing countries⋆^☆^^☆☆^

**DOI:** 10.1016/j.ijedudev.2021.102350

**Published:** 2021-04

**Authors:** Maryam Akmal, Lant Pritchett

**Affiliations:** a Center for Global Development, 2055 L St NW, Washington, DC 20036, United States; b Blavatnik School of Government, University of Oxford, Radcliffe Observatory Quarter, 120 Walton St, Oxford, United Kingdom

**Keywords:** Learning assessments, Education quality, Human capital, Equity, Wealth

## Abstract

Whereas the MDG was a simple schooling goal the Sustainable Development Goals (SDGs) have a number of targets for learning. Target 4.1 specifies not just that all children complete primary and secondary school but that this schooling leads to “relevant and effective learning outcomes” and Indicator 4.1.1 tracks progress goal using the proportion of children reaching “minimum proficiency” at early (grade 2/3), intermediate (primary complete) and late (end if lower secondary) stages of basic education with the aim that "all youth…achieve literacy and numeracy" (Target 4.6). We use the Annual Status of Education Report (ASER) data from India and Pakistan, and Uwezo data from Kenya, Tanzania, and Uganda that assess all children in a given age range, whether in school or not, on simple measures of learning in math, reading (local language), and English, to quantify how much achieving within country *equality* between the richest 20 percent and the poorest 40 percent in (a) grade attainment and (b) learning achievement by grade would contribute to an SDG-like global *equity* goal of universal numeracy and literacy for all children by age 12−13. We have three empirical findings. First, except in Kenya equalizing grade attainment between children from rich and poor households would lead to only modest progress in achieving universal numeracy, closing only between 8% (India) and 25 % (Pakistan) of the existing gap to universal literacy. Second, equalizing the learning profiles, that is, closing the gap in learning for children in the same grade between those from the poorest 40 percent of households and the richest 20 percent, would close between 16 % (Pakistan and Uganda) and 34 % (India) of the gap to universal numeracy, and between 13 % (Uganda) and 44 % (India) of the gap to universal literacy. Third, even with complete equality in grade attainment and learning achievement with children from the richest 20 percent children from poorer households still be far from the equity goal of universal numeracy and literacy, as even children from the richest 20 percent of households are far from universal mastery of basic reading and math by ages 12−13. In the currently low performing countries achieving universal literacy and numeracy to reach even a minimal proficiency of global equity goal will require more than just closing the rich-poor learning gap, it will take progress in learning for all.

## Highlights

• The paper quantifies the extent to which achieving within country equality would bring countries closer to global equitygoals.
• Equalizing grade attainment between rich and poor children leads to only modest progress.
• Equalizing learning profiles results in larger gains that vary across countries.
• Even with equality of schooling and learning, children are still far from mastering basic reading and math by 12−13.
• Reaching equity goals will require more than closing rich-poor gaps, but progress in learning for all.

## 1 Introduction

A self-ordained professor’s tongue too serious to fool

Spouted out that liberty is just equality in school

"Equality," I spoke the word as if a wedding vow

Ah, but I was so much older then, I’m younger than that now

Bob Dylan, My Back Pages

The drive to universal enrollment drive of the past few decades has been enormously successful at getting children to school. In many countries, at the margin the beneficiaries of expanding enrollment were traditionally marginalized groups such as children from poorer households, girls, and rural dwellers. In India the DHS data show the fraction of girls aged 12−13 years from the poorest 40 percent of households enrolled in school increased from 32.0%–86.5% from 1992/1993–2015/2016, whereas, since rich males of the same age had an enrollment rate of 95.2 percent in 1992/93 there was obviously little additional gain. In Uganda DHS data show an increase in the enrollment among poorer girls from 55.2 % in 1995 to 87.4 % in 2011.^1^ Millions of children remain out of school (Global Education Report Monitoring Team, 2017) and eliminating remaining inequalities in enrollment and grade attainment across household income, parental characteristics, sex, ethnicity, residence, and disability is essential.

Achieving universal enrollment and grade completion will not be sufficient to achieve the “minimum proficiency” learning goals and targets envisioned in SDG Goal 4 (World Bank, 2018). Whelan (2014) estimates that 96 % of children around the world receive some schooling, but only 37 % achieve basic learning by the end of primary school. Spaull and Taylor (2015), using data from Southern and East Africa Consortium for Monitoring Educational Quality (SACMEQ), show that 53 % of Ugandan children were innumerate at age 12 but only 4% had never enrolled in school, 14 % had enrolled in Grade 1 but dropped out before age 12, and 33 % of all children aged 12 had completed Grade 6 but were nevertheless innumerate. Recent data show large gaps from “minimum proficiency” in numeracy and literacy both early (grade 2/3) and late (age 15). The recent report of the PAL network using the ICAN (Internationally Comparable Assessment of Numeracy) instrument that uses face to face oral methods that do not require reading or writing to answer numeracy questions to assess early grade numeracy. They find across districts (not nationally representative) in thirteen countries that only between .4 percent of 29 percent of children in grade 2/3 can do a simple subtraction problem (PAL Network, 2019). The PISA-D report covered only seven countries and showed the proportion of the 15 year olds in school and assessed *not* reaching PISA level 2 in reading was 78.5 percent for boys and 75.1 percent for girls and the percent *not* reaching PISA level 2 in mathematics was 86.3 percent for boys and 89.9 percent for girls.^2^ Crouch et al. (2017, 2020) call the exclusion of most children from achieving competence in literacy and numeracy an “equity crisis” on a global scale.

How much would efforts to achieve *equality* in measures of learning outcomes across groups within a country help achieve a global equity goal based on a “minimum proficiency” in learning? It would certainly help some: one of the best documented facts about schooling around the world is that both grade attainment (Filmer and Pritchett, 2001, among many others) and assessed learning at any given grade or age^3^ tends to be lower for children from poorer or less advantaged households. Hanushek and Woessmann (2011) argue that in empirical analyses of the learning of children in the same grade in a given country a measure of household SES is often the single biggest factor explaining learning differences, a correlation that operates through numerous causal channels^4^ . Achieving equality of opportunity and outcomes in grade attainment (access, repetition, drop-out) and in learning at each grade for the disadvantaged and marginalized is clearly a major concern for education systems.

Yet assessments across the developing world show that absolute learning levels are low across the board, for children from both rich and poor households. In the average of the seven PISA-D countries the fraction of the most advantaged quartile *not* reaching PISA level 2 was 74.7 percent for math and 58.3 percent for reading^5^ . According to ASER data for 2015, by age 12 only half of the children from richer households can do a basic division problem. This is 20 percentage points more than the figure for children from poor households, but still 50 percentage points away from universal mastery of a very basic arithmetic operation. If even children from richer households have low learning levels, then raising the learning of disadvantaged children to that of the privileged may still leave them well short of absolute learning levels needed for global equity. Global equity will require more than equalization of the poor to the level of the rich within each country. Here paper we ask: “how much more than within country equality is needed to achieve a global equity goal of minimum proficiency?”

We use grade attainment and learning profiles by wealth constructed from ASER and Uwezo data to quantify how much learning would change under various counter-factual scenarios:

• How much would the likelihood that a child from a poor household is literate (defined as the ability to read a short, second grade level, story) or numerate (defined as the ability to do a simple division problem) change if they had the same grade attainment as a child from a rich household while keeping their existing learning profile?
• How much would the likelihood that a child from a poor household is literate or numerate improve if they had the learning profile of a child from a rich household while keeping their existing grade attainment profile?
• If children from poor households had exactly the same learning *and* grade attainment as children from rich households, how far would they be from achieving universal literacy or numeracy?

## 2 Data and methods

### 2.1 Sampling

ASER (meaning “impact”), an annual household-based survey of the reading and arithmetic learning of children, has been carried out in India and Pakistan. Uwezo (meaning “capability”), an ASER- like survey in Africa, has been carried out in Kenya, Uganda, and Tanzania. This data has four features not often found together. One, all children aged 5–16 are in the sampling frame, not just those in a given grade or enrolled in school. Two, in each country all children are assessed using the same instrument and hence performance (of a very simple sort) can be compared across age and grade.^6^ Three, whether or not the child is currently enrolled, the child’s highest grade of enrollment are reported. Four, there is some data on assets that can be used to construct a proxy for the wealth of each child’s household. A downside of the ASER data is that they are representative only of rural districts of India and ASER Pakistan includes rural and only some urban districts (Table 1).

**Table 1:** Number of Survey Years and Total Assessed Children, by country.

Country	Years	Number of Children Tested in One or More Subjects
India	2009−2014	2.9 million
Kenya	2009, 2011−2015	0.7 million
Pakistan	2012−2015	0.8 million
Tanzania	2010−2015	0.5 million
Uganda	2010−2015	0.4 million

We combine the data from each country across all available years. The resulting data set contains grade attainment information for approximately 5.7 million children. Of the total children, math test results are available for 5.2 million children, local language reading results are available for 4.8 million children, and English reading results are available for 3.7 million children. Table 2 shows the how children who took at least one test across the five countries.

**Table 2:** Percentage of All Sampled Children by Household Wealth Category.

Country	Bottom 40 percent of Households	Middle 40 percent of Households	Top 20 percent of Households
India	44.25	38.64	17.11
Kenya	42.52	40.53	16.94
Pakistan	41.53	39.66	18.80
Tanzania	39.59	40.96	19.44
Uganda	38.37	41.32	20.31

### 2.2 Learning measure

The ASER-style assessments are meant to be extremely simple to implement and understand and the trade-off is that the resulting learning measure is also simple. For reading, there is a single card (in the child’s preferred language) which is used for all ages. This card contains letters, words, a short sentence (Grade 1 level), and a short passage of one or two paragraphs (Grade 2 level).^7^ Each child’s performance is categorically coded by the highest level they are comfortable doing: "nothing" is level 1, "recognize letters" is level 2, "read words" is level 3, "read sentence" is level 4, and "read Grade 2 paragraph" is level 5.^8^ These levels are categories, not cardinal numbers.^9^ We create a binary indicator that is 1 for "level 5″ and zero for all other categories call that an indicator of minimum proficiency for a 12/13 child in “literacy.”

Similarly, the math assessment is a single card that contains a collection of one-digit numbers, some two-digit numbers, some subtraction problems of two-digit numbers (requiring "carry"), and division problems of dividing a one-digit number into a three-digit number with a remainder (e.g., 824/6, 517/4).^10^ Again this is categorically coded and we use just a binary indicator for "level 5″ as our definition of minimal proficiency in numeracy.^11^

Patel and Sandefur (2020) create a “Rosetta Stone” link between various international assessments such as TIMSS, PIRLS, PASEC, LLECE, and ASER by asking a sample of children in Bihar, India to sit a test that includes items from the various assessments using the Non-Equivalent Groups with Anchor Test (NEAT) approach. The authors found that it was difficult to estimate precisely a concordance of ASER to international assessments as the top-coded category of ASER (what we use as our indicator of minimum proficiency) is near the bottom of these assessments. For instance, the distribution of TIMSS scores of children with an ASER level of 5 on math would have a mean of 406 with an inter-quartile range over 100 points, so a quarter of all children with a “top code” on ASER would be below 300 on a TIMSS-like assessment of their overall mathematics competence. This is a very large dispersion on TIMSS of children with the same ASER score as, since 5 is the top-coded level in ASER even children with (much) higher math capability than "do division" are included in this category with those for whom “do division” this is their highest level of capability. Similarly, an ASER level 5 on reading is equivalated by NEAT to PIRLS score of 418, with an inter-quartile range of 74. For our purposes the point is that, if TIMSS and PIRLS scores are roughly the equivalent of PISA, then about half of children reaching an ASER/Uwezo level 5 would be below the level 2 level of performance of PISA. Even the top-coded level of ASER-like assessments is a low bar for “minimal proficiency” for literacy and numeracy.

### 2.3 Construction of the asset index

We create an asset index as a measure of household wealth. As in Filmer and Pritchett (2001) the weights on the individual assets (e.g. “own a TV”) or housing characteristics (e.g. “solid house”) are produced using principal components analysis (PCA). There are up to 17 asset/housing indicators across the surveys, but different countries have different data and the PCA is done separately for each country, using only those asset indicators that have fewer than about 10 % missing values, and so each country’s index has different weights.^12^ We use the household asset index score to assign a *unique* rank to each household,^13^ divide the data into deciles and assign households to a wealth group, the top 20 percent as “richer”, the bottom 40 as poorer, and the middle 40 percent. Each child is assigned their respective household’s wealth status. Because there can be more children in poorer households there are not exactly 40 percent of the children in the poorest 40 percent of households and Table 2 shows the distribution of children across the different wealth categories as the “children from the bottom 40 percent of households” is roughly, but not exactly “the bottom 40 percent of children by HH wealth.”

Filmer and Pritchett (2001) argue that the asset index is a superior measure of long-run household SES for predicting education outcomes than consumption or income as its components are more accurately measured and it is more stable. The asset index is quire reliable particularly for assigning households to broad groups. Table 3 shows the scoring factors from PCA for the nine asset variables used for Kenya (Appendix C shows the same table for the other countries). The wealth categories produce very sharp separation between the bottom 40 percent and top 20 percent. In the top wealth group 83 percent have electricity and 88 percent a house with a wall whereas this is only 1 percent and 5 percent for households in the bottom 40 percent. The richest 20 percent and poorest 40 percent by these asset groups are very different groups economically, that is, these are not fine distinctions between households that would be “observationally equivalent” to a casual observer but are found in the data, but are big, obvious gaps in households economic and, one would guess, social status and condition^14^ .

**Table 3:** Scoring Factors and Summary Statistics for Variables Entering the Computation of the First Principal Component: Kenya.

Asset	Scoring Factors	Mean	SD	Scoring Factors X SD	Mean Poorest 40 percent	Mean Middle 40 percent	Mean Richest 20 percent
Bicycle Available	0.15	0.26	0.44	0.07	0.09	0.39	0.38
Car Available	0.24	0.03	0.18	0.04	0.00	0.01	0.16
Electricity Available	0.45	0.20	0.40	0.18	0.01	0.16	0.83
House with Wall	0.39	0.37	0.48	0.19	0.05	0.49	0.88
Mobile Available	0.34	0.64	0.48	0.16	0.38	0.84	0.96
Motorbike Available	0.21	0.07	0.25	0.05	0.00	0.07	0.23
Radio Available	0.30	0.66	0.47	0.14	0.41	0.82	0.92
TV Available	0.47	0.19	0.39	0.18	0.00	0.12	0.86
Water Available	0.28	0.41	0.49	0.14	0.22	0.47	0.76

*Note:* Each asset ownership/housing characteristic variable takes the value 1 if true, 0 otherwise. Scoring factor is the "weight" assigned to each variable in the linear combination of the variables that constitute the first principal component.

While we refer to the top 20 percent as “rich” we hope to not confuse this with the “richest” few hundred households in a country or even the “elite” (for example, the top 0.1 % or 1%) who would, in any case, be hard to capture in a survey (and, for a rural based sample like India, would mostly be excluded in any case). But our calculations are robust to narrower definitions of “the rich”, Appendix D shows that changing the threshold of “the rich” from 20 percent to 15, 10, or even 5 percent does not substantially alter the percent literate or numerate among the “rich” at each grade level and hence changing this threshold will not affect by much the overall simulation results.

## 3 Learning profiles of the rich and the poor

Progress towards goals for universal achievement of a learning goal can be tracked using the progress on a learning measure of an age cohort, as this combines the results of grade progression and learning per grade. Using the ASER/Uwezo data set we can produce descriptive *learning profiles* by age and by household wealth. These show the proportion of children of a given age and household SES who are literate or numerate. In the Section 4 we decompose these wealth gaps in literacy and numeracy by age into grade attainment by age and wealth (how much behind "grade for age" are children from poor households) and into a learning profile by grade (how likely are children from poor households in each grade to be less literate or numerate than children from richer households). Section 5 does counter-factual calculations of how much of the gap from universal attainment of literacy or numeracy for children from poor households can be erased if they achieve the same grade progression, learning profile by grade, or both, as children from rich households.

**Fig. 1:**
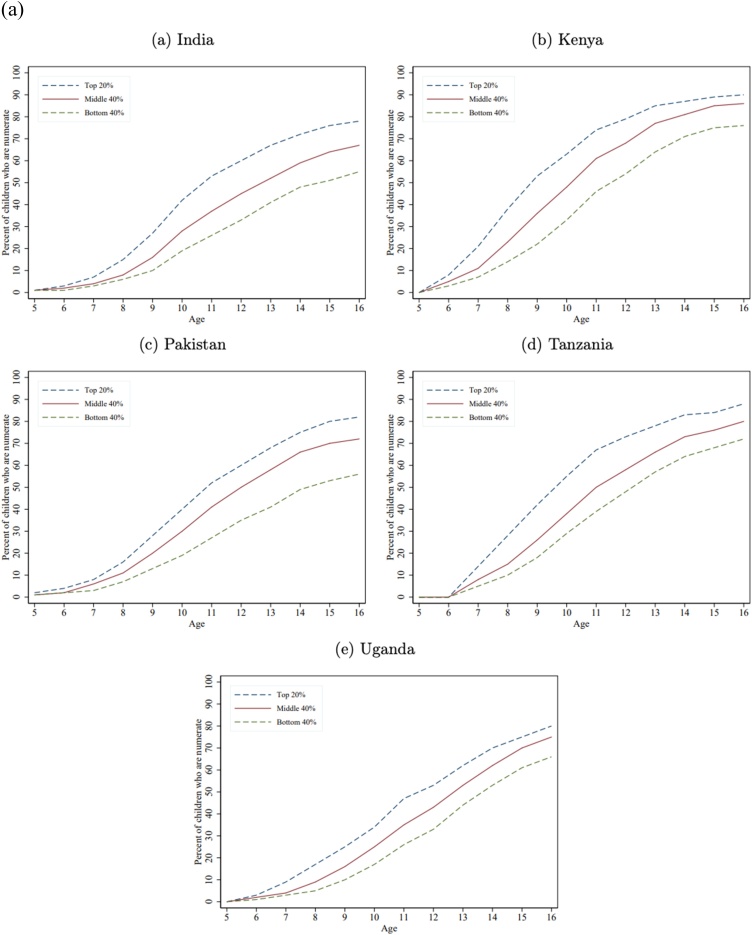

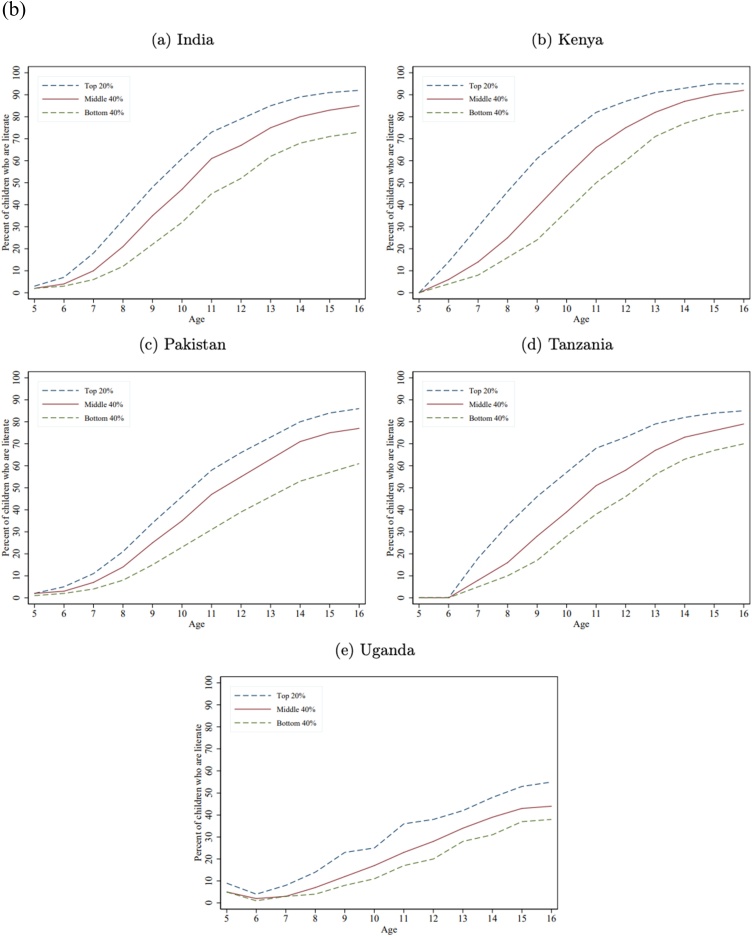

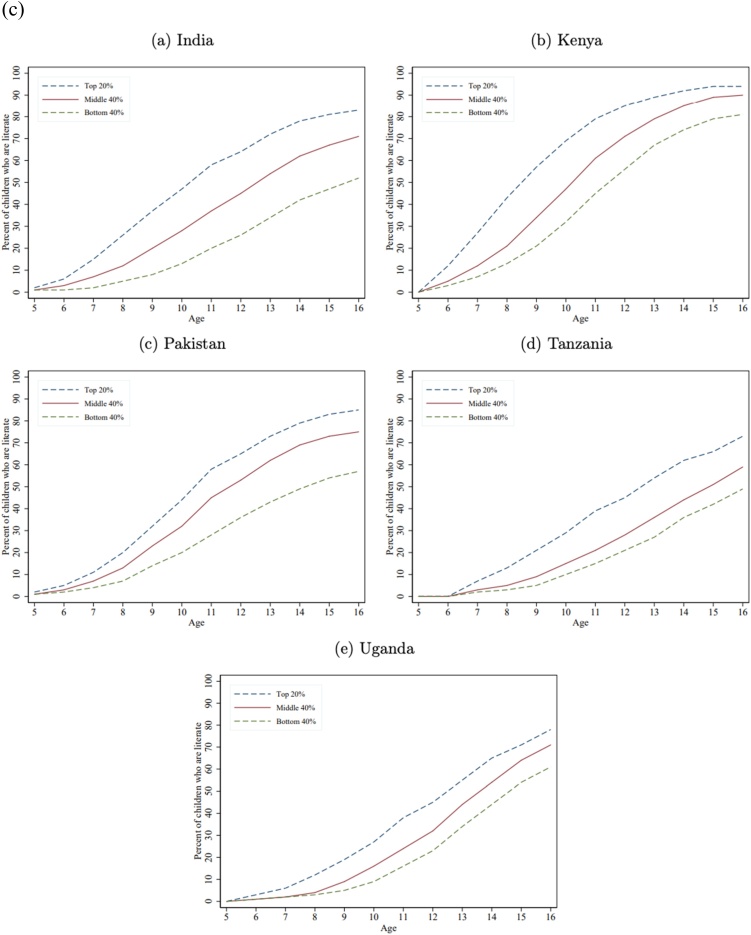
a) Learning Profiles by Age and Household Wealth: Math. b) Learning Profiles by Age and Household Wealth: Local Language. c) Learning Profiles by Age and Household Wealth: English.

Fig. 1a) shows the fraction of children in each wealth group who are numerate (able to solve a simple division problem). Not surprisingly, as division is a relatively late curricular concept, the learning trajectory gaps by wealth only begin to emerge after age 7, as essentially none of the children can do division at ages 5 or 6^15^ . But wealth gaps in numeracy do emerge very early, often are large by age 8, and become very large by age 12 and persist as the children get older (in some cases the gaps diminish but this is a result of a very low bar for “minimum proficiency” and does not indicate that the gaps in a broader, not top-coded, measure of mathematics proficiency is not growing wider).

Fig. 1b) shows the learning profile by age for literacy, defined as reading a Grade 2 level paragraph in the local language across the five countries. The results are similar to numeracy: with very small differences at very young ages, wealth gaps emerging by ages 7–8, and large gaps across wealth groups by ages 12−13, that persist as the children get older.

While mastery of English is not a fundamental skill nor SDG goal, like numeracy and literacy, we also show those results as all five countries do assess mastery of English and because one expect that the wealth gaps are different for a more “advanced” skill and one that likely to be important as a marker of status. Fig. 1c) shows differences in learning emerge by ages 7–8 and grow by ages 12−13. The gap in literacy between children from rich and poor households tends to be above 20 percentage points, except in India where it is almost double that. Kenya tends to do better on English than Uganda and Tanzania, which could be reflective of the fact that in Kenya English tends to be the medium of instruction as early as Grade 1 (Trudell, 2016) whereas in Tanzania, Kiswahili is the primary language of instruction (Bashir et al., 2018) and in Uganda, English is the language of instruction only from Grade 4 onward (Trudell, 2016).

Table 4 shows the gap at age 12 between children from the richest and poorest households for numeracy, literacy (local language), and English, which reveals two key facts.

**Table 4:** Gaps in numeracy, literacy, and English between the richest 20 percent and poorest 40 percent at age 12.

Country	Numeracy (percent who can do simple division)				Local language literacy (percent who can read a simple grade 2 level story)				English			
	Richest 20 percent	Poorest 40 percent	Wealth Gap (richest less poorest) (sorted on this column)	Gap of richest 20 percent to universal (100 percent)	Richest 20 percent	Poorest 40 percent	Wealth Gap (richest less poorest)	Gap of richest 20 percent to universal (100 percent)	Richest 20 percent	Poorest 40 percent	Wealth Gap (richest less poorest)	Gap of richest 20 percent to universal (100 percent)
India	60	33	27	40	79	52	27	21	64	26	38	36
Kenya	79	54	25	21	87	60	27	13	85	56	29	15
Pakistan	60	35	25	40	66	39	27	34	65	36	29	35
Tanzania	73	48	25	27	73	46	27	27	45	21	24	55
Uganda	53	33	20	47	38	20	18	62	45	23	22	55
Median	60	35	25	40	73	46	27	27	64	26	29	36

First, in all countries except Kenya and in all the three domains the wealth gap in achieving “minimum proficiency” at age 12 between children between the richest 20 percent and poorest 40 percent of households is over 20 percentage points. The median gap is 25 percent for numeracy, 27 percent for literacy, and 29 percent for English. India has the largest wealth gap for all three learning measures: at age 12, the percentage of Indian children from rich households who can do basic math is roughly twice that of the children from poorer households. Alcott and Rose (2017) use ASER data from India and find wealth gaps of similar magnitude. It is clear that any plan for reaching universal proficiency will have to close the very large gaps between the rich and poor.

The second fact apparent in Table 4 is that the absolute level of numeracy among the rich is typically far from universal and varies a great deal across countries. In Kenya 79 percent of children from rich households can do division, but in India and Pakistan only 60 percent of children from rich households can, and in Uganda, the figure is only 53 percent. This finding implies that if we think global equity requires achieving a minimal level of numeracy for all children, even children from the richest quintile in Pakistan and Uganda, and rural India, are only just past half-way to this global equity goal. While reaching universal will require addressing the wealth gaps, addressing the wealth gaps alone is far from sufficient, given that even the richest 20 percent are far from receiving a minimally adequate education.

## 4 Decomposing the learning trajectory by age into grade attainment and grade-based learning profiles

The learning gap between children from rich and poor households at each age can be decomposed into differences in grade attainment and differences in learning achievement by grade, the *descriptive* learning profile. The grade attainment profile is the fraction of children of a given age in any given group (where a group could denote rich/middle/poor, girl/boy, urban/rural, maternal/paternal education, state/region, etc.) who have completed a particular grade. The *descriptive grade-based learning profile* is the share of children with a particular grade attainment who are literate or numerate (and could be any other measure of learning). For any age cohort and for any given group, the fraction of the group who are literate is just the grade attainment weighted average of the grade-based learning profile. Therefore, mechanically, a group could have higher literacy or numeracy because either (1) the group on average had higher grade attainment, or (2) the group has a steeper learning profile so that a child from one group is more likely to be literate or numerate in any given grade, or (3) both (Pritchett and Sandefur, 2017, who carry out this same decomposition for male/female gaps).

This simple decomposition corresponds, not surprisingly, to two difference approaches to closing learning gaps between groups. One set of policies that expand the grade attainment of the lagging group (e.g., scholarships, conditional cash transfers, etc.). These policies often do not address learning profile differences, for instance, policies of automatic grade promotion advance all students and hence increase grade attainment, whether or not the students have learned the age/grade appropriate material. A second set of policies address the learning profiles for the disadvantaged (e.g., teaching at the right level, early childhood programs that focus on school readiness, etc.).

We look at the grade attainment and learning achievement profiles for children aged 12-

13. While grade attainment profiles have generally improved over time because of higher enrollments, there is no reason to assume that learning profiles have improved as well. For instance, the ASER results from India in 2014 show learning profiles have worsened at times (ASER, 2014). From 2010–2014, the percentage of Grade 5 students who could read a simple story fell from 54 % to 48 %, and the percentage of Grade 5 students who could do a simple division problem fell from 36 % to 26 % (ASER, 2014)^16^ .

The decomposition of the age learning profile into grade attainment and learning achievement per grade is based on some simple equations. The fraction of an age cohort that is at minimum proficiency (literate or numerate) is given by Eq. (1):

(1) $${\text{\%}MP}_{WG,D}=\sum_{g=0}^{g=G}\alpha_{WG}^{g}\text{*}s_{WG,D}^{g}$$

Where $\alpha_{WG}^{g}$ is the share of children in wealth group WG (rich20, poor40, middle40) whose highest grade was *g* and $s_{WG,D}^{g}$ is the share of children from wealth group WG who reached minimum proficiency in domain area D (numeracy, literacy, English) if their highest grade was g.

Using these simple equations we can calculate various hypothetical scenarios of: (1) equal grade attainment between rich and poor, (2) equal learning achievement at a given grade of rich and poor, or (3) both^17^ .

The equal grade attainment scenario we calculate what the learning levels of children from the poorest 40 percent of households would be if they had the same grade attainment as children from the richest 20 percent of households ($\alpha_{Rich}^{g}$) but still had their own existing learning profiles ($s_{Poor,D}^{g}$):

(2) $${\text{\%}MP}_{Poor,D}^{GradeattainmentofRich}=\sum_{g=0}^{g=G}\alpha_{Rich}^{g}\text{*}s_{Poor,D}^{g}$$

The equal learning profile scenario calculates how much higher literacy/numeracy would be for children from the poorest households if they retained their existing grade attainment profiles ($\alpha_{Poor}^{g})$ but had the grade-based learning profiles of children from the richest 20 percent of households ($s_{Rich,D}^{g}$):

(3) $${\text{\%}MP}_{Poor,D}^{LearningprofileofRich}=\sum_{g=0}^{g=G}\alpha_{Poor}^{g}\text{*}s_{Rich,D}^{g}$$

Just from examining Eqs. (1) to (3) one can see that the gain from the improvement in learning profiles of children from poor households is going to be larger the higher their grade attainment and the larger the gap in the learning profile between poor and rich.

### 4.1 Grade attainment profiles

**Fig. 2:**
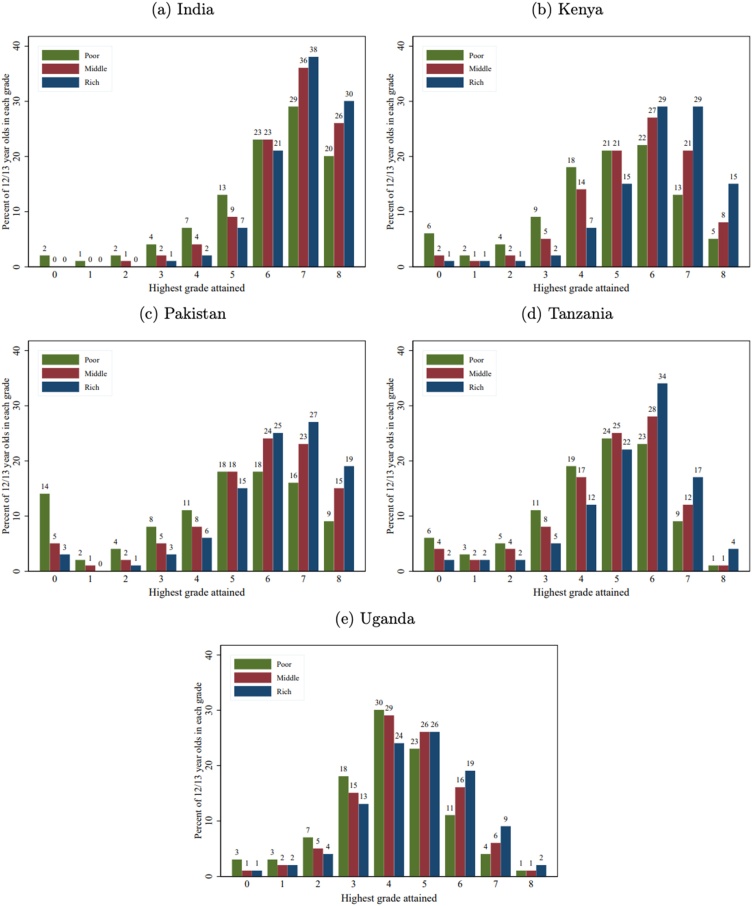
Grade Attainment by Wealth, Ages 12-13.

Fig. 2 shows the grade attainment profiles for 12−13 year old children using the highest grade attained for both those still in school and those who have dropped out, dis-aggregated by wealth.18 If children were to start at age 6 and progress one grade per year, one would expect most 12−13 year old children to be in Grades 7–8. But we know from previous analysis of enrollment and attainment profiles that there is substantial late enrollment, grade repetition, and drop-out and these are more likely for children from poorer households. Hence, there is a spread in grade attainment. For the African countries, we see more bunching in the middle (Grades 4–6), which is particularly pronounced for Uganda. However, one can see that children from rich households are more likely to have reached the "age-appropriate" Grades 7–8. For example, in Tanzania, 17 % of 12−13 year old children from richer households are in Grade 7 compared to 9% of those from poorer households. Note that in India, a substantially higher proportion of 12−13 year old children are in the "age-appropriate" Grades 7–8, almost certainly reflecting higher transition to lower secondary, likely in part from policies of automatic promotion. Note that the proportion of "never enrolled" is higher for children from poor households, but above 10 % only in Pakistan. Therefore, the grade deficit for children from poor households aged 12−13 is mostly late enrollment and lower grade progression rather than the fact these children never enroll in school.

### 4.2 Descriptive grade-based learning profiles

**Fig. B2:**
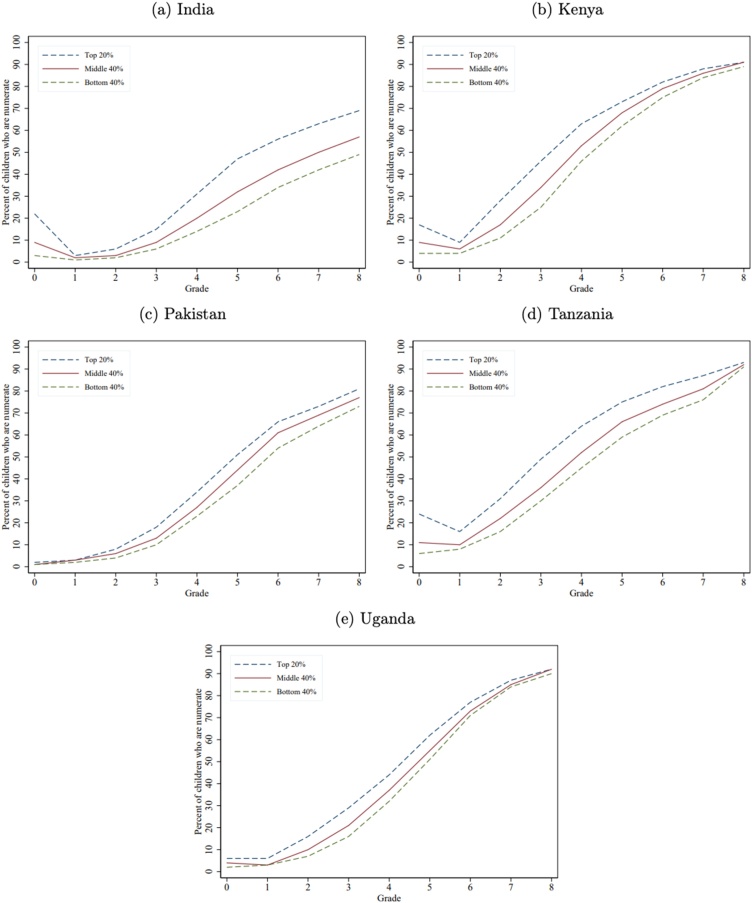
Learning Profiles by Wealth: Math (All Ages).

**Fig. B3:**
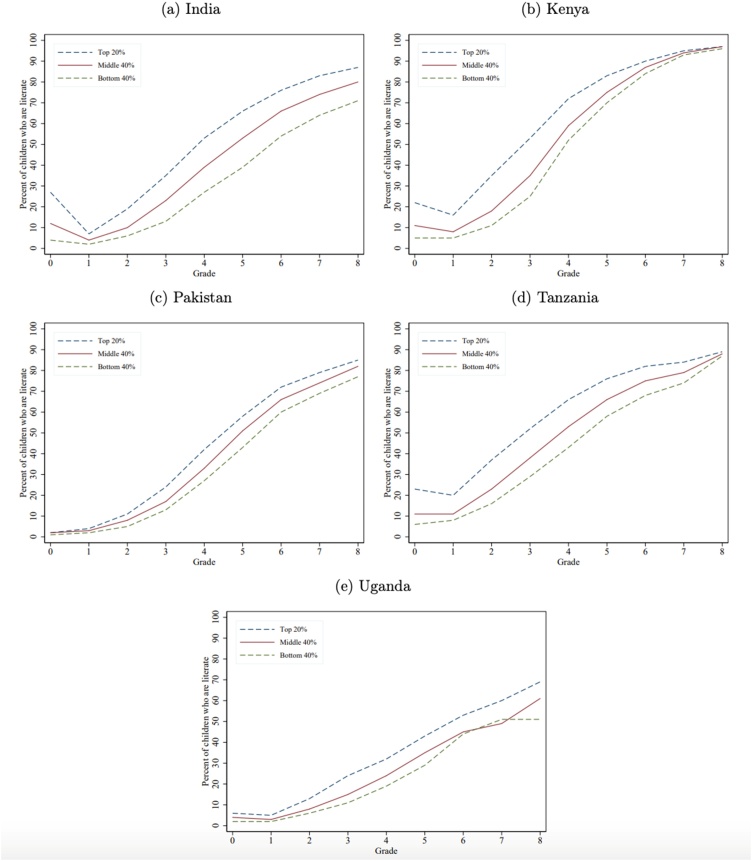
Learning Profiles by Wealth: Local Language (All Ages).

**Fig. B4:**
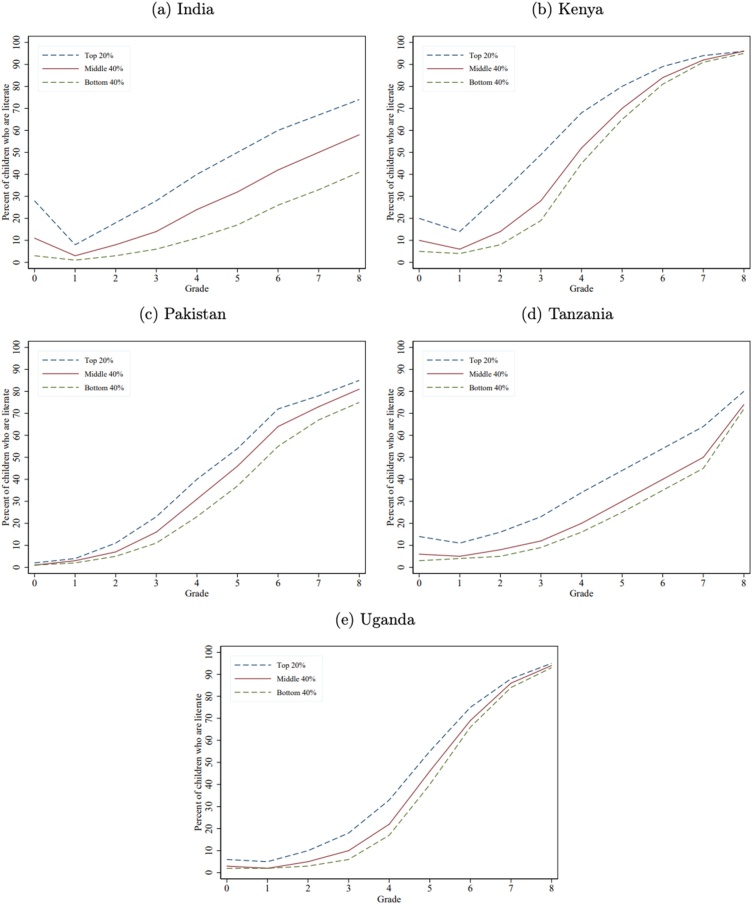
Learning Profiles by Wealth: English (All Ages).

The grade-based learning profiles in Fig. B2, Fig. B3, Fig. B4 in the appendix show that in India, even after 8 years of formal schooling, there is a large, steady, and persistent gap in

basic numeracy and local language literacy between children from rich and poor households. The gap emerges early, as found in a study by Alcott and Rose (2017). In Pakistan, the gap in learning between children from rich and poor households is smaller (compared to India) but also emerges early and remains steady through to Grade 8.

In the African countries, we see the gap emerging early, remaining largely steady over the years and then mostly closing by Grade 8. This is possibly due to the fact that children with lower performance do not advance in grades and hence the difference in the descriptive learning profile is due to selection as children from poorer households repeat grades or drop-out, not learning. Moreover, as our measure of numeracy is top-coded at a low level, this says nothing about the evolution of the overall gap in terms of a more sophisticated measure of mastery of a broader learning domain called mathematics—children from rich households may be getting further and further ahead on a different measure of mathematics competency. Work by (Das et al., 2020) Das, Singh and Chang (2018) shows that test score gaps that have developed by Grade 3 remain steady over primary school years and then widen dramatically by the time these children reach age 17 due to differential dropouts: children from poor households drop out at higher rates than children from rich households. While low-performing children from rich households may stay in school, even the high-performing children from poor households tend to drop out.

### 4.3 Counter-factual calculations

We run various simulations to see how total learning levels would change under different scenarios of grade attainment and learning achievement levels for children from poor households^18^ . As we emphasize below in section 4.4 these are not “projections” as they use descriptive, not causal relationships and hence are illustrative.

#### 4.3.1 What if all children from poor households have the grade attainment profiles of children from rich households?

In the first hypothetical scenario, we explore what happens to learning levels if children from poor households had the grade attainment profiles of children from rich households, while keeping their existing learning profile (learning levels by grade). Such a scenario would still leave more than 40 percent of Indian, Pakistani, and Ugandan children from poor households innumerate and unable to read a simple English story. In India, such a hypothetical scenario represents a gain of mere 5 and 4 percentage points in numeracy and English literacy respectively—only covering less than 10 % of the gap between current learning levels of children from poor households and the goal of universal literacy/numeracy. However, in Kenya, where learning per grade is relatively high, such a scenario would cover close to half the gap from universal literacy in local language and English.

#### 4.3.2 What if all children from poor households have the same learning profiles by grade as children from rich households?

In the second hypothetical scenario, we test what happens if all children from poor households have the learning profiles of children from rich households while maintaining their current grade attainment levels. A hypothetical scenario where all children from poor households aged 12−13 suddenly have the learning profiles of children from rich households (while keeping their current grade attainment profiles) would still leave more than 40 percent of the children from poor households innumerate in India, Pakistan, and Uganda. In Tanzania, more than half the children from poor households will still be unable to read a simple English story—with less than one-fourth of the gap from universal literacy being covered. In Uganda, more than half the children from poor households will still be unable to read a simple story in their local language—with only 13 % of the gap from universal literacy being covered. This means that for most countries in our data set, a significant proportion of the children will be left illiterate and innumerate even if the learning gap between the rich and the poor was completely closed. However, excluding Kenya and Pakistan, improving learning profiles often covers a larger share of the gap between current learning levels of the poor and the goal of universal learning compared to improving grade attainment profiles.

The gains in learning depend on the initial levels of illiteracy and innumeracy among children from poor households. For example, India has high illiteracy and innumeracy among children from poor households. The learning gap between children from the richest and poorest households is also huge: 27 percentage points in math, 26 percentage points in local reading, and 39 percentage points in English. A combination of a large number of illiterate/innumerate children from poor households and a big learning gap between the richest and poorest households leads to a significant jump in literacy/numeracy under the scenario where children from poor households have learning profiles of children from rich

households: a jump of 22 percentage points in math, 20 percentage points in local reading, and 34 percentage points in English. Despite these jumps, close to a quarter of the children from poor households remain unable to read a simple sentence. On the other hand, for low illiteracy/innumeracy (among children from poor households) countries such as Kenya, the hypothetical scenario of giving poor children the learning profiles of children from rich households leads to an improvement in learning of 12, 13, and 15 percentage points for math, local reading, and English respectively. For such countries the percentage of children from poor households who are illiterate and innumerate is relatively low, so there isn’t much gain to be made.

#### 4.3.3 Counter-factual Scenario 3: what if all children from poor households have the grade attainment and learning profiles as children from rich households?

**Fig. 3:**
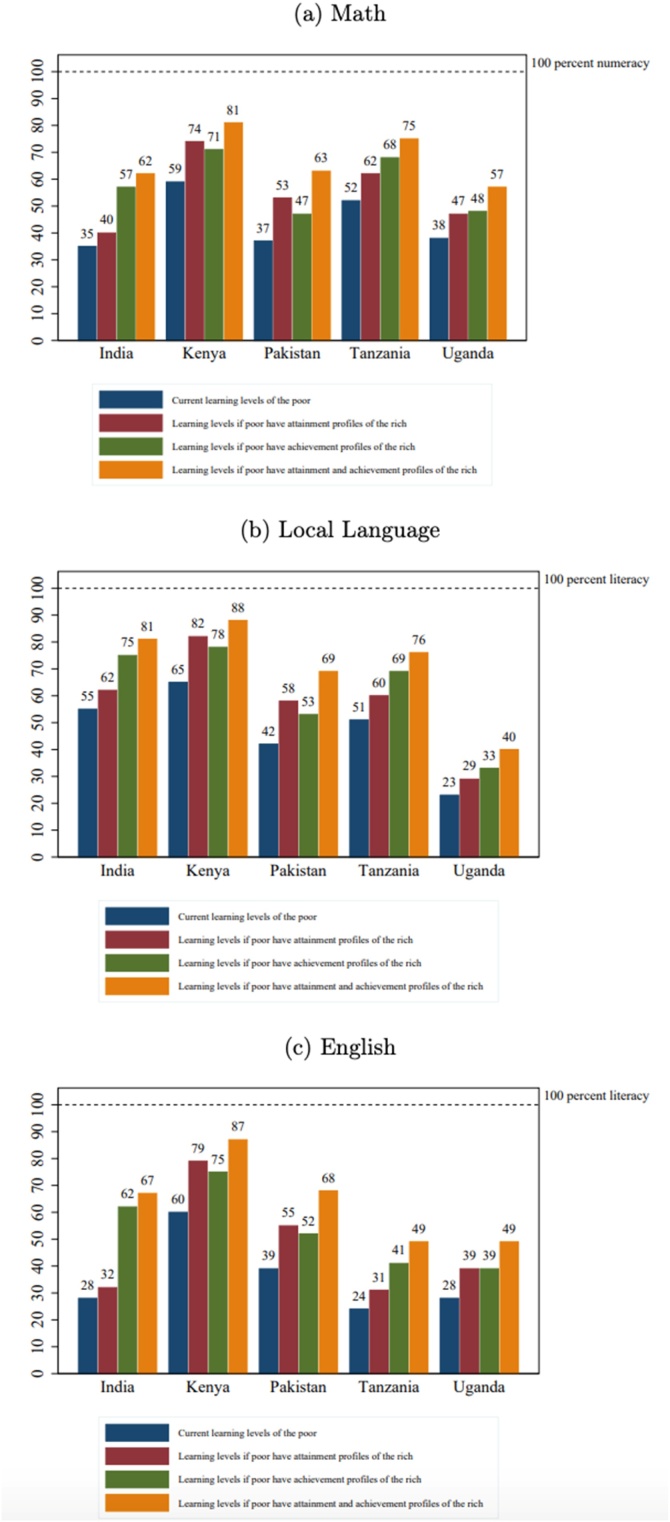
Counter-factual Simulations.

In the third hypothetical scenario, we explore what happens to learning levels if we completely close the gap between children from rich and poor households, that is, all poor children have the grade attainment and learning achievement profiles of children from rich households. For all countries except Kenya, bringing the learning and grade attainment levels of children from poor households to the levels of children form rich households still brings us nowhere close to the goal of universal mastery of basic literacy and numeracy. A hypothetical scenario where all children from poor households aged 12−13 suddenly have the learning profiles and grade attainment profiles of children from rich households would still leave more than one-third of the children from poor households innumerate in India, Pakistan, and Uganda—with less than half of the gap to universal numeracy being covered. In Tanzania, more than half the children from poor households will still be unable to read a simple English story. In Uganda, more than half of the children from poor households will still be unable to read a simple story in their local language. This means that for most countries in our data set, a significant proportion of the population will still be illiterate and innumerate even if the learning and grade attainment gap between the rich and the poor was completely closed (Fig. 3).

**Fig. 4:**
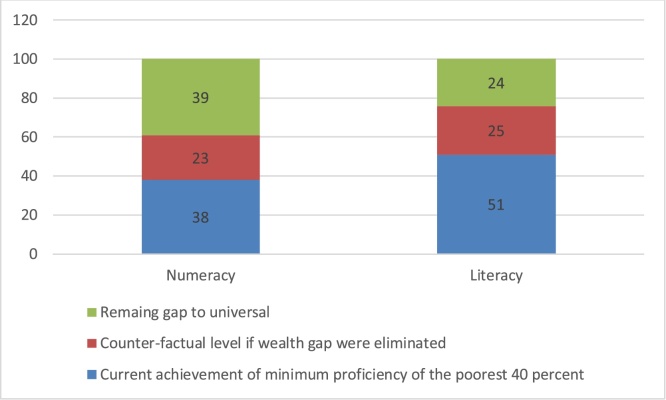
The complete elimination of the wealth gap in achievement of minimum proficiency in numeracy and literacy of children 12-13 still would still leave a large gap to achieving universal targets.

Table 6 and Fig. 4 are a different way of summarizing the results of Table 5 for the two key indicators of numeracy and literacy. Instead of the gains from wealth gap equalization in (a) grade attainment, (b) learning profile and (c) both (full learning achievement equalization) in percentage points gains for the poorest 40 percent of the 12−13 aged cohort it shows the same in terms of the achieving something like SDG indicator 4.1.1 of universal minimal proficiency. So, for instance, the proportion 12−13 year olds in the ASER-Pakistan sample who are numerate is 37 percent. The gains from equalizing the grade attainment of the poorest 40 percent with richest 20 percent is 16 percentage points (Table 5) and the gap to universal is 63 percentage points (= 100−37) so the poor-rich equalization of grade attainment eliminates 25 percent of the gap (= 16/63).

**Table 5:** Results of counter-factual simulations of equalizing the grade attainment of rich and poor, the learning profile of rich and poor, or both.

Country	Numeracy				Literacy				English			
	Gains from equalizing grade attainment of poor to rich	Gains from equalizing the learning profile of poor to rich	Gains if the poor had the same learning outcomes as the rich	Deficit from universal achievement of minimum proficiency even if outcomes of poor are equalized to the rich (percentage points)	Gains from equalizing grade attainment of poor to rich (percentage point)	Gains from equalizing the learning profile of poor to rich (percentage points)	Gains if the poor had the same learning outcomes as the rich	Deficit from universal achievement of minimum proficiency even if outcomes of poor are equalized to the rich	Gains from equalizing grade attainment of poor to rich (percentage point)	Gains from equalizing the learning profile of poor to rich (percentage points)	Gains if the poor had the same learning outcomes as the rich	Deficit from universal achievement of minimum proficiency even if outcomes of poor are equalized to the rich (percentage points)
India	5	22	27	38	7	20	26	19	4	34	39	33
Kenya	15	12	22	19	17	13	23	12	19	15	27	13
Pakistan	16	10	26	37	16	11	27	31	16	13	29	32
Tanzania	10	16	23	25	9	18	25	24	7	17	25	51
Uganda	9	10	19	43	6	10	17	60	11	11	21	51
Median	10	12	23	37	9	13	25	24	11	15	27	33

**Table 6:** For the typical country, closing the grade attainment gap between poorer and richer children aged 12-13 would only close 20 percent of the gap of the poorest to universal minimum proficiency and even closing the entire wealth gap in learning achievement would only close about half of the gap to a global equity goal of universal minimum proficiency in numeracy and literacy.

Country	Numeracy				Literacy in a local language			
	Current percent of children from the poorest 40 percent of households reaching minimum proficiency	Percent of the gap from existing level of the poorest 40 percent to universal (100) percent achievement of minimum proficiency from equalizing with the richest 20 percent on:			Current percent of children from the poorest 40 percent of households reaching minimum proficiency	Percent of the gap from existing level of the poorest 40 percent to universal (100) percent achievement of minimum proficiency from equalizing with the richest 20 percent on:		
		Grade Attain- ment	Learning Profile	Both (Learning Achievement)		Grade Attain- ment	Learning Profile	Both (Learning Achievement)
India	35	8	34	42	55	16	44	58
Kenya	59	37	29	54	65	49	37	66
Pakistan	37	25	16	41	42	28	19	47
Tanzania	52	21	33	48	51	18	37	51
Uganda	38	15	16	31	23	8	13	22
Median	38	21	29	42	51	18	37	51

This leads to three main facts (including facts about counter-factuals as facts)

First, except for Kenya, the past gains in enrollment of the poor mean there just isn’t that much progress left to be made by grade attainment gains and hence equalizing the grade attainment of rich and poor through age 12−13 would for the typical (median) country eliminate only about 20 percent of the gap to universal minimum proficiency (21 percent of numeracy, 18 percent for literacy). This is not to say efforts to reach universal completion of (at least) lower secondary are not important as this is an independent SDG and basic education has long been seen as a basic human right and the equalization of poor and rich around access and grade completion is an important target in its own right for many reasons. But, however important reaching universal completion is, it will not be enough to close much of the remaining gaps to global equity of reaching universal minimal learning goals as most of the learning deficit is of children who are already in school.

Second, closing the wealth gaps in learning is frequently as important—or much more important—than closing the gaps in grade attainment. In India (where grade promotion is automatic) the gains from grade attainment are small(ish) but the gains from equalizing learning of poor and rich are quite large (34 percent of the gap to universal for numeracy, 44 percent for literacy). So, if rather than just being carried through grades there was attention to the learning of children from poorer households massive progress could be made. This however varies from country to country as in Pakistan the learning profiles of the rich are not that much better than the poor so the grade attainment equalization gains are larger than those from learning profile equalization.

Third, even if the learning achievement gap were completely closed between poorer and richer, this would only close about half or less of the gap to universal minimum proficiency, for the simple reason that even children from the richest 20 percent of households are quite from universally reaching even the low thresholds of numeracy (being able to do a simple division problem) or literacy (being able to read a simple grade 2 level paragraph). This is in spite of the fact that most children from richer households are in grades 6/7/8 (or higher) by ages 12−13. For countries to reach universal minimum proficiency in numeracy and literacy the learning profiles (gains in learning per grade) have to be much, much, steeper than they are, even for the better off children.

### 4.4 Limitations and caveats

As with any descriptive data, there are major limitations to our counter-factual calculations and we stress these are not “projections” or “forecasts” but are merely illustrative of the features of the existing ASER/Uwezo data. There are three significant weaknesses of our calculations.

First, the main limitation of our counter-factual simulations is that we assume that the increase in learning from one grade to the next that a child would experience if they were enrolled is the average of those children that do enroll (of the same category, e.g. rich or poor). This assumes that the increments to learning in the descriptive learning profile by grade is something like a constant Local Average Treatment Effect (LATE). The calculations assume that if a child who dropped out in Grade 4 had persisted to Grade 5, their likelihood of gaining literacy or numeracy in that year would be equal to the average observed gain in literacy from Grade 4 to Grade 5 of those that did enroll. This assumption is likely false because of the positive self-selection of students into further grades, or in other words, those students who drop out in earlier grades are likely to be those were lower in achievement distribution and would gain less in learning from one year to the next. This positive self-selection of students implies that at least part of the gain in the descriptive learning profile does not reflect causal learning because those with higher cumulative learning persisted in school. If this is true then all our counter-factual simulations overstate gains in learning. Since the descriptive learning profile is steeper than the causal learning profile, our estimates are optimistic and expansion of schooling may produce even less literacy than we suggest. Kaffenberger and Pritchett (2020) show in a formal model of pedagogical processes that produce learning profiles that this over-estimation of the gains to learning goals from enrollment expansion could be very large.

Second, our assumption of a constant learning profile that an expansion of schooling does not cause the learning profile to deteriorate for all students. Again, if expansions tend to produce flatter learning profiles for all students (not just the change in the selection effect indicated above) then this *inflates* our estimates of the counter-factual for learning gains from higher grade attainment (Pritchett and Sandefur, 2017).

Third, our counter-factual simulations of equalizing the learning profile by a measure of socio-economic status might by *very* counter-factual, in the sense that it is not obvious a set of policy measure or interventions exist that would accomplish this equalization. In the 2015 PISA results *all* OECD countries had a PISA score gap between the best and worst quartiles of their Economic Social and Cultural Status index of at least 50 points (on an assessment normed to a standard deviation across OECD students of 100). Even in countries with a traditional of low social inequality, strong education systems and high absolute levels of available resources (e.g. Finland, Norway, Denmark, Japan, etc.) there is a large (over 50 points) gap between the lowest and highest quartile. Therefore, assuming the developing countries—with less resources, lower capability generally and generally weaker education systems could eliminate the SES gap in learning profiles entirely is an illustrative, rather than likely or even plausible, counter-factual. We present these results in the sense of “even if a country could do this” rather than as a “realistic” alternative.

## 5 Conclusion

This paper adds to the literature on equity gaps in cumulative learning by using data that allows us to document the roles of both grade attainment and of learning per grade, whereas most data sources do not have learning assessment results for out of school children and hence either can show wealth gaps in schooling and grade attainment or wealth gaps in the learning of the enrolled, but not both. We are able to quantify how much of the learning gap between children aged 12−13 from rich and poor households is due to a grade completion disadvantage versus a shallower learning profile (less learning per grade).

The results confirm with these five countries, two South Asian (India, Pakistan) and three East African (Kenya, Tanzania, Uganda) several important points relative to attaining the SDG goals for achieving universal minimal proficiency.

First, given the past successes in expanding enrollments and grade attainment have closed much of the grade attainment gap to universal completion the remaining wealth gaps are relatively small (in these countries) and hence the progress to universal minimum proficiency to be had from eliminating wealth gaps in grade attainment is relatively modest—about 20 percent of the gap.

Second, even when the poorest are in school their learning progress tends to be slower than for the richer children. Closing these learning profile gaps is potentially important—there is nearly always more progress from achieving equalization in learning profiles by wealth than in equalizing grade attainment.

Third, relative to global standards, even of minimum proficiency, learning levels are low for children in these countries from richer and poorer households. The learning levels for children from richer households (top 20 percent) are low relative to minimum proficiency. In order to make significant gains in improving literacy levels, closing the learning achievement and grade attainment gaps of children from rich and poor households is a positive step but far from sufficient. For the world to get closer to the goal of universal literacy, *all* children across *all* wealth groups (and other indicators of advantage and disadvantage) will have to experience sustained dramatic gains in learning.

## CRediT authorship contribution statement

**Maryam Akmal:** Conceptualization, Methodology, Formal analysis, Writing - original draft. **Lant Pritchett:** Conceptualization, Methodology, Formal analysis, Writing - original draft.

## Figures and Tables

**Table A1:** Score Conversions.

Country	Year	Uwezo (Original)	ASER (Equivalent)
Kenya	2009, 2015	7 (divide)	5 (divide)
Kenya	2011−2014	8 (divide)	5 (divide)
Tanzania	2015	9 (multiply)	5 (divide)
Tanzania	2010, 2011−2014	7 (multiply)	5 (divide)
Uganda	2010−2015	7 (divide)	6 (divide)

**Fig. B1:**
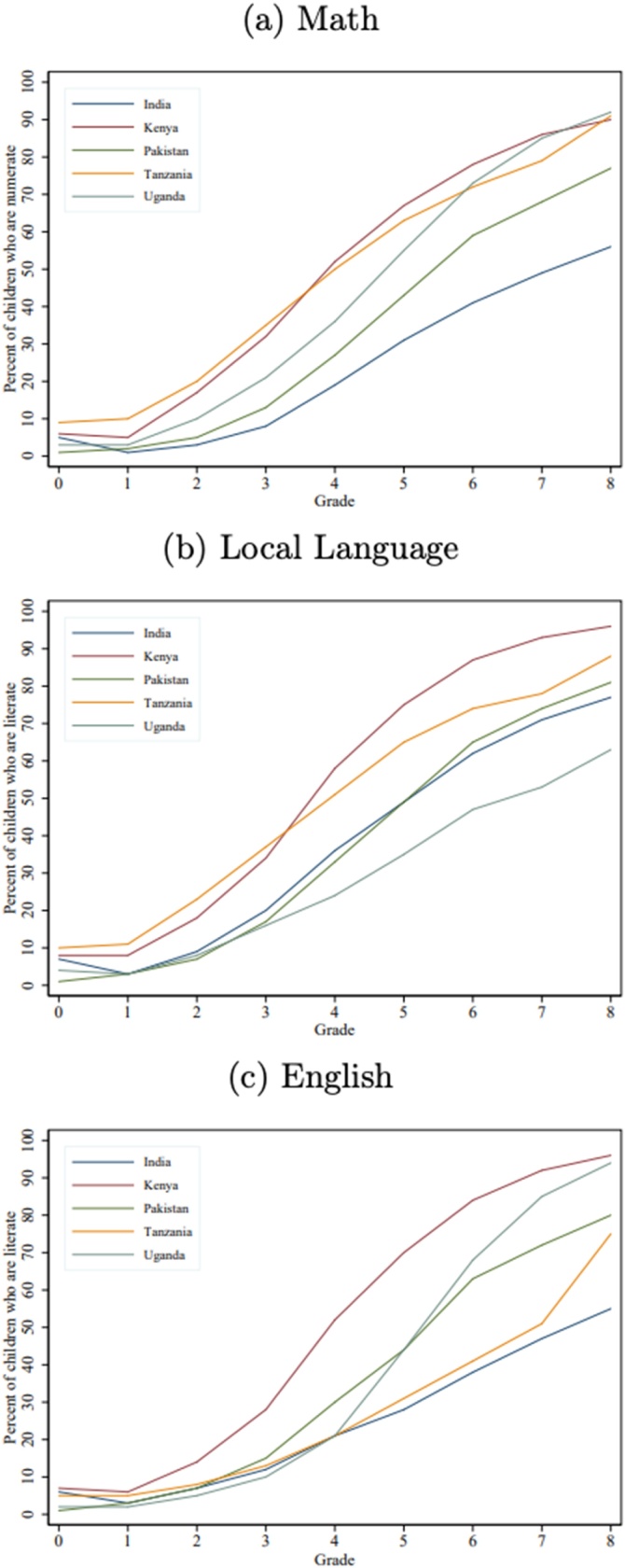
Learning Profiles (All Ages).

**Table C1:** India.

Asset	Scoring Factors	Mean	SD	Scoring Factors * SD	Mean Poorest 40%	Mean Middle 40 %	Mean Top20 %
Electricity Available	0.45	0.74	0.44	0.20	0.44	0.96	1.00
Mobile Available	0.40	0.68	0.47	0.19	0.43	0.82	1.00
Solid House	0.40	0.35	0.48	0.19	0.09	0.36	0.95
TV Available	0.53	0.50	0.50	0.26	0.03	0.79	1.00
Toilet Available	0.45	0.42	0.49	0.22	0.11	0.51	1.00

**Table C2:** Pakistan.

Asset	Scoring Factors	Mean	SD	Scoring Factors *SD	Mean Poorest 40%	Mean Middle 40 %	Mean Top20 %
Electricity Available	0.51	0.88	0.33	0.17	0.71	1.00	1.00
Mobile Available	0.53	0.81	0.39	0.21	0.54	1.00	1.00
Own House	0.15	0.91	0.28	0.04	0.87	0.92	1.00
Solid House	0.36	0.30	0.46	0.17	0.08	0.22	1.00
TV Available	0.55	0.59	0.49	0.27	0.10	0.91	1.00

**Table C3:** Tanzania.

Asset	Scoring Factors	Mean	SD	Scoring Factors * SD	Mean Poorest 40 %	Mean Middle 40 %	Mean Top20 %
Bicycle Available	0.08	0.49	0.50	0.04	0.38	0.58	0.53
Car Available	0.27	0.02	0.15	0.04	0.00	0.00	0.12
Electricity Available	0.47	0.24	0.42	0.20	0.01	0.20	0.81
House with Wall	0.36	0.44	0.50	0.18	0.15	0.53	0.85
Mobile Available	0.35	0.57	0.49	0.17	0.24	0.76	0.92
Motorbike Available	0.28	0.09	0.29	0.08	0.00	0.07	0.33
Own Cattle	−0.08	0.30	0.46	−0.04	0.33	0.30	0.24
Own Sheep	−0.12	0.34	0.47	−0.06	0.38	0.35	0.23
Radio Available	0.29	0.63	0.48	0.14	0.32	0.79	0.90
TV Available	0.51	0.15	0.35	0.18	0.00	0.03	0.71

**Table C4:** Uganda.

Asset	Scoring Factors	Mean	SD	Scoring Factors * SD	Mean Poor-est40 %	Mean Middle40 %	Mean Top20 %
Bicycle Available	0.20	0.51	0.50	0.10	0.30	0.65	0.62
Electricity Available	0.50	0.11	0.32	0.16	0.00	0.02	0.53
Mobile Available	0.45	0.59	0.49	0.22	0.09	0.89	0.94
Motorbike Available	0.35	0.10	0.30	0.11	0.00	0.02	0.46
Radio Available	0.36	0.66	0.47	0.17	0.39	0.81	0.88
TV Available	0.48	0.07	0.26	0.12	0.00	0.00	0.34
Water Available	0.16	0.30	0.46	0.07	0.21	0.33	0.47

*Note:* Each variable takes the value 1 if true, 0 otherwise. Scoring factor is the "weight" assigned to each variable in the linear combination of the variables that constitute the first principal component.

**Table D1:** Sensitivity Test for Percent Numerate Among the Rich: Math (India).

Wealth	0	1	2	3	4	5	6	7	8
Top 20 %	21.56	2.55	5.93	15.32	31.35	46.84	56.30	62.94	69.44
Top 15 %	21.67	2.53	5.97	15.78	31.69	47.19	56.75	63.17	69.67
Top 10 %	20.85	2.57	6.18	16.07	31.77	47.50	56.56	63.48	69.89
Top 5%	18.65	2.64	6.07	16.04	32.30	47.48	57.06	63.72	69.76

**Table D2:** Sensitivity Test for Percent Literate Among the Rich: Local Language (India).

Wealth	0	1	2	3	4	5	6	7	8
Top 20 %	27.23	7.24	18.73	34.96	52.61	65.92	76.16	82.53	87.07
Top 15 %	26.18	7.43	19.07	35.52	53.16	66.10	76.47	82.54	87.19
Top 10 %	25.56	7.58	18.92	35.62	53.09	66.13	76.43	82.44	87.34
Top 5%	23.03	7.59	18.62	35.70	53.15	66.36	77.10	82.72	87.54

**Table D3:** Sensitivity Test for Percent Literate Among the Rich: English (India).

Wealth	0	1	2	3	4	5	6	7	8
Top 20 %	28.50	7.61	17.96	28.58	39.82	50.12	60.29	67.59	73.95
Top 15 %	28.21	7.84	18.24	29.31	40.28	50.39	60.57	67.72	74.03
Top 10 %	27.01	7.70	18.00	30.14	40.15	50.76	60.42	67.76	73.54
Top 5%	23.43	7.87	18.32	31.07	41.06	51.00	61.24	67.33	73.12

## Appendix A Score conversions for categories of mathematics

Table A1

## Appendix B Grade Learning Profiles for Richest 20 percent and Poorest Children

Fig. B1

## Appendix C Scoring Factors and Summary Statistics for Variables Entering the Computation of the First Principal Component

Table C1, Table C2, Table C3, Table C4

## Appendix D Sensitivity to Definition of “rich” households for India

We conduct a sensitivity test to see if the percent literate or numerate at each grade level changes drastically when we change the cut-off defining our top wealth group. It could be argued that the “top 20 percent” is too broad of a category to pick up true levels of inequality. We do the sensitivity test for India, the country with the largest sample size in our data. The following tables show the percent literate and numerate (for all ages) at each grade level for the top 20 percent, top 15 %, top 10 %, and top 5%. The results show that the percent literate or numerate remains roughly constant across the various cut-offs, showing that the top 20 percent is a reasonable cut-off to define the richest group for the purposes of studying wealth gaps in learning.

Table D1, Table D2, Table D3.

## Footnotes

1 These are from the Filmer and Pritchett (2001) asset index. The data cited are from the World Bank education attainment website that shows grade attainment and enrollment by asset index, sex, and urban/rural residence.

2 PISA-D results, Table 12 and Table 33.

3 See, among many other sources, the household SES differences reported in PISA (2015) using their Economic, Social and Cultural Status indicator or the World Development Report on Education (2018)

4 The association between a child’s learning achievement and household SES can be causally mediated by many factors, such as poor nutrition (Alderman and Bundy, 2012), parental education (Dubow et al., 2009) and attention (Davis-Kean, 2005), and stress factors (Lupien et al., 2000). Analysis by Paxson and Schady (2007) in Ecuador shows that children from wealthier households and more educated parents have higher test scores. This association grows stronger as children grow older, implying that there is an increasing gap in test scores between children from rich and poor households with age. Furthermore, positive sorting between households and schools can further aggravate inequalities: richer households are able to select better schools (Anand et al., 2019).

5 PISA Results, Tables 14 and 35.

6 It is technically very demanding to create an instrument that is equated (say, using IRT) to be comparable and covers a large array of grades, which is why it is rarely done. ASER-like surveys use the same instrument, at the cost of having lots of “bottom-coding” for young children and “top-coding” for older children and not being able to make fine distinctions.

7 ASER data for India for years 2010, 2011, and 2013 do not contain information about reading in English. However, the "local" language test was sometimes administered in English. We classify such instances as actually testing literacy in English. Uwezo data for Uganda for years 2010 and 2011 do not contain any information about testing literacy in the local language.

8 ASER data for India and Pakistan codes literacy in English slightly differently from literacy in local language: "nothing" is level 1, "recognize capital letters" is level 2, "recognize small letters" is level 3, "read words" is level 4, and "read sentences" is level 5. Uwezo data from Kenya, Tanzania, and Uganda codes literacy in local language and in English using the same five step classification for both languages.

9 A cardinal measure would imply that the gap between 1 and 2 is the same as between 2 and 3 or 3 and 4 and that therefore the gap between 1 and 3 was twice that as between 1 and 2 (or 2 and 3). None of this can be asserted of the ASER/Uwezo levels (which could just as easily, and perhaps more accurately, just assigned letters (e.g. level A, B, C, etc. or arbitrary names (e.g. Red, Blue, Green)). But, the same is true of all other assessments, including the numbers produced by more sophisticated methods like Item Response Theory (IRT). Many assessments produce scores that appear, and are often treated as if they were, cardinal, e.g. that the gap between a country scoring 380 and 400 is “the same” as the gap between 480 and 500. But IRT scores across students (and therefore, across groups) are only unique up to a monotone transformation (Ho, 2016), and are more properly treated as ordinal, as any set of numbers that preserves the *relative* rankings of students represents equally well their performance on answering any given set of questions.

10 The Uwezo instrument in Kenya and Uganda have division of a two-digit number by a one-digit number.

11 Uwezo tests are mostly coded on a 1−5 scale. However, for certain years and subjects in Kenya, Tanzania, and Uganda, Uwezo tests are coded on a 1−7 or a 1−9 scale. In order to facilitate comparisons across countries and different years, we ensure that the highest order skill measured denotes literacy and/or numeracy. For numeracy, the highest order skill tends to be division, and for literacy, it tends to be the ability to read a simple story. More details about how we re-calibrate those tests to align with the standard 1−5 scale used for ASER can be found in Table A1 in the appendix. While streamlining the ASER and Uwezo scores does not allow perfect comparability across countries (for example, in Tanzania, multiplication rather than division tends to be highest skill measured on math tests), the conversions ensure that the highest order skill measured by each test gets assigned a 5. Hence, we classify as literate or numerate any individual who is in the top-coded category for that country.

12 The data for Uganda (2011) contained a few observations with different assets for children in the same household. This problem affected a minor number of observations for asset variables denoting access to electricity, TV, mobile, radio, bicycle, bike, and water. To resolve this data anomaly, if one of the children in the same household is assigned as having a certain asset, we assume all the remaining children in the household also have access to that particular asset. This problem was present for fewer than 4% of the observations for each affected asset variable in the Uganda (2011) sample.

13 While assigning unique values is potentially problematic because households with the same asset index may be assigned different rank values, unique values allow creating wealth groups such that the top 20% wealth group has approximately 20% of the population, the middle 40% has approximately 40% of the population, etc. Particularly when there are few assets (e.g. India has only five assets) there will be a large number of ties for household asset index score. This makes it impossible to create such proportional cut-offs without assigning unique values. This is innocuous for our analysis as (1) since the households that were tied were indistinguishable, any assignment of households into the two adjacent categories (e.g., bottom 40 versus middle 40) would have produced the same expected value of results, and (2) since we are, in this paper, only comparing poorest 40 percent and richest 20 percent there obviously were no ties across those categories.

14 These type of asset indices are widely used and the principal components analysis typically produces a first factor with a large eigen value and that “explains” around 30 percent of the total variance across all assets.

15 That the gaps emerge late demonstrates we are measuring a learned/acquired skill and not measuring some notion of an “intrinsic” child aptitude or potential or “intelligence” except insofar as these are realized in an acquired and demonstrable skill/capability/competence.

16 This is just used as an example that there is no general evidence that descriptive learning profiles are improving over time and that there are documented instances of decline (on specific measures) in specific countries and periods. Johnson and Parrado (2020) compare assessments for India and find suspiciously large differences across states in the different measures.

17 We could do this same decomposition for the middle 40 percent as well to get the whole population effect of closing wealth gaps but instead focus on the poorest 40 percent. The results are consistently ordered between poor, middle, rich so that the results are predictable.

18 In the counter-factual calculations use grade-based learning profiles for 12 to 13 year old children only whereas the graphs in Fig. B2, Fig. B3, Fig. B4 in the appendix show grade-based learning profiles for children of all ages.
